# Identifying data gaps in early childhood physical activity evidence

**DOI:** 10.3389/fped.2024.1485500

**Published:** 2024-11-21

**Authors:** Jessica Wimberly, Aleah Nguyen, Erica Memoli, Matt Kasman, Bill Heerman, Russell Pate, Evan Sommer, Adam Sedlak, Lydia Reader, Ross A. Hammond, Shari Barkin

**Affiliations:** ^1^ Department of Pediatrics, Virginia Commonwealth University/Virginia Commonwealth University Health System, Richmond, VA, United States; ^2^ Center on Social Dynamics and Policy, Brookings Institution, Washington, DC, United States; ^3^ Department of Pediatrics, Vanderbilt University Medical Center, Nashville, TN, United States; ^4^ Arnold School of Public Health, University of South Carolina, Columbia, SC, United States; ^5^ Division of Computational and Data Sciences, Washington University in St Louis, St Louis, MO, United States

**Keywords:** physical activity, children, socioeconomic status, contributors, self-efficacy, screen time

## Abstract

Childhood physical activity sets the foundation for health. While we know many factors that contribute to physical activity, there are limitations in our knowledge, especially in early childhood. Through our review, we identify gaps in existing datasets to guide future research.

## 1 Introduction

Increasing childhood physical activity (PA) promotes both short- and long-term health outcomes. PA improves cardiometabolic health, bone strength, mood, memory, learning, motor skills, executive function, language, and numeracy (1–5). Preschool children are at a key period for motor, emotional, and cognitive development, all of which are bolstered by achieving recommended amounts of PA (2, 4, 5). These early benefits extend into adolescence and adulthood to support long-lasting health benefits such as a decreased risk of cardiovascular disease, certain cancers, diabetes, metabolic syndrome, obesity, high cholesterol, insulin resistance, and increased adiposity (6, 7).

The U.S. Department of Health Physical Activity Guidelines for Americans recommends that children ages 3–5 years-old engage in 180 min/day of total PA, at least 60 min of which must be moderate-vigorous physical activity (MVPA) (8). Unfortunately, most young children do not meet the recommended PA guidelines. Evidence shows that nearly half of preschool children do not engage in the recommended levels of PA and thus are at greater risk for adverse health outcomes (9, 10). As adverse health outcomes increase in prevalence, understanding the factors that affect children's engagement in PA becomes increasingly important (7).

Below is a brief review of existing early childhood PA research. We identify a few common data gaps and provide suggestions to guide future research on early childhood PA.

## 2 Childhood physical activity

PA incorporates levels of intensity categorized as light, moderate, and vigorous. Most literature focuses on MVPA as it is more closely associated with health outcomes than total PA (11). PA is typically measured with subjective tools like MVPA recall, physical activity questionnaires, or through objective tools like accelerometers or pedometers (12). It can take preschoolers up to 11 h, within a 24-h period, to achieve the daily recommended amount of MVPA as they often engage in PA in sporadic, short bursts, making quantitative measures of PA over a long-time frame especially valuable. These activity spurts last anywhere from seconds to minutes in duration and comprise 75% of MVPA by preschool children (13).

We use the socioecological model to organize the current state of understanding of contributors to early childhood PA behaviors (14). At the individual level, age, sex, ethnicity, body mass index (BMI), and socioeconomic status (SES) influence PA (15–17). As children age, their PA levels decrease, and the rate of decline in PA is greater in younger children than adolescents (15, 17). Around the age of 3, research shows that young children spend more time being physically active rather than sedentary, but at the age of 4, their activeness declines by nearly half (18). This decrease in PA continues from ages 5–7 but trends rather gradually, indicating a slower decline (18). Factors which explain these trends have not been identified as behavioral and lifestyle factors were not simultaneously assessed, leaving gaps in the data. PA also varies by sex. Male children achieve up to 2.27 min/h more total PA than females at all ages and significantly higher levels of MVPA (15, 19). Current research notes ethnic minorities in the United States to be less physically active than White populations of the same age (20). Additionally, children with a higher BMI typically engage in less MVPA than children with a lower BMI (21). Lastly, SES shapes PA behaviors. Children with lower SES spend less time engaging in PA for leisure and, when physically active, it is less vigorous than higher SES children of the same age (22).

At the intrapersonal level, factors such as parent belief that PA is important, support and engagement in PA, and peer support of PA impact childhood PA. Preschool-aged children are especially susceptible to social influence at the family level. Parent engagement with their children in PA correlates consistently with increased child PA. Preschool children are 5.8 times more likely to engage in PA if parents engage in PA (23). Active fathers have a greater impact than active mothers on PA. Preschool children with active mothers are twice as likely to engage in PA, while children with active fathers are 3.8 times more likely (24). Preschool-aged children who have peers that engage in, and support PA, are more physically active (24). This, in part, is due to the increased socialization and companionship experienced in the presence of peers, a child's personality traits and their personal qualities (25). Active children tend to find peers with similar levels of activeness, and the converse is true as well. However, structured social interactions between peers with different personality traits can help stimulate a child's PA regardless, indicating that the presence of peers is a key contributor to children's PA (25).

At community and environmental levels of impact, neighborhood safety and built environment, school environment, community resources, as well as local and state policy influence childhood PA. In the home, children with access to more PA resources such as portable play equipment, including bikes and jump ropes, meet guidelines for PA on more days than those with fewer resources (26). Parent perceptions of neighborhood safety mediate access to outdoor resources and child PA. If parents perceive neighborhoods as unsafe, their children engage in less indoor and outdoor PA (27). However, in other studies parent perception of neighborhood crime negatively impacts only parents’ vigorous PA but does not affect their children's (28). Community resources, such as playgrounds, green spaces and parks, and activity trails support PA in children. In fact, MVPA levels are 43% higher when children are outdoors vs. indoors (29, 30). Lower SES neighborhoods often have fewer PA resources than higher SES neighborhoods (26). In one study, access to playgrounds with “high” environment scores (more trees, open play spaces, and shrubbery) increased step counts in preschoolers (31). In another study assessing preschool environment quality, researchers found that preschools with higher quality scores, larger playgrounds, and portable playground equipment were associated with children who engaged in more MVPA (32). PA-related policies also impact preschool-aged children's PA; increased recess duration and decreased playground density increase total daily PA in males and recess-based PA in females (33).

**Figure 1:**
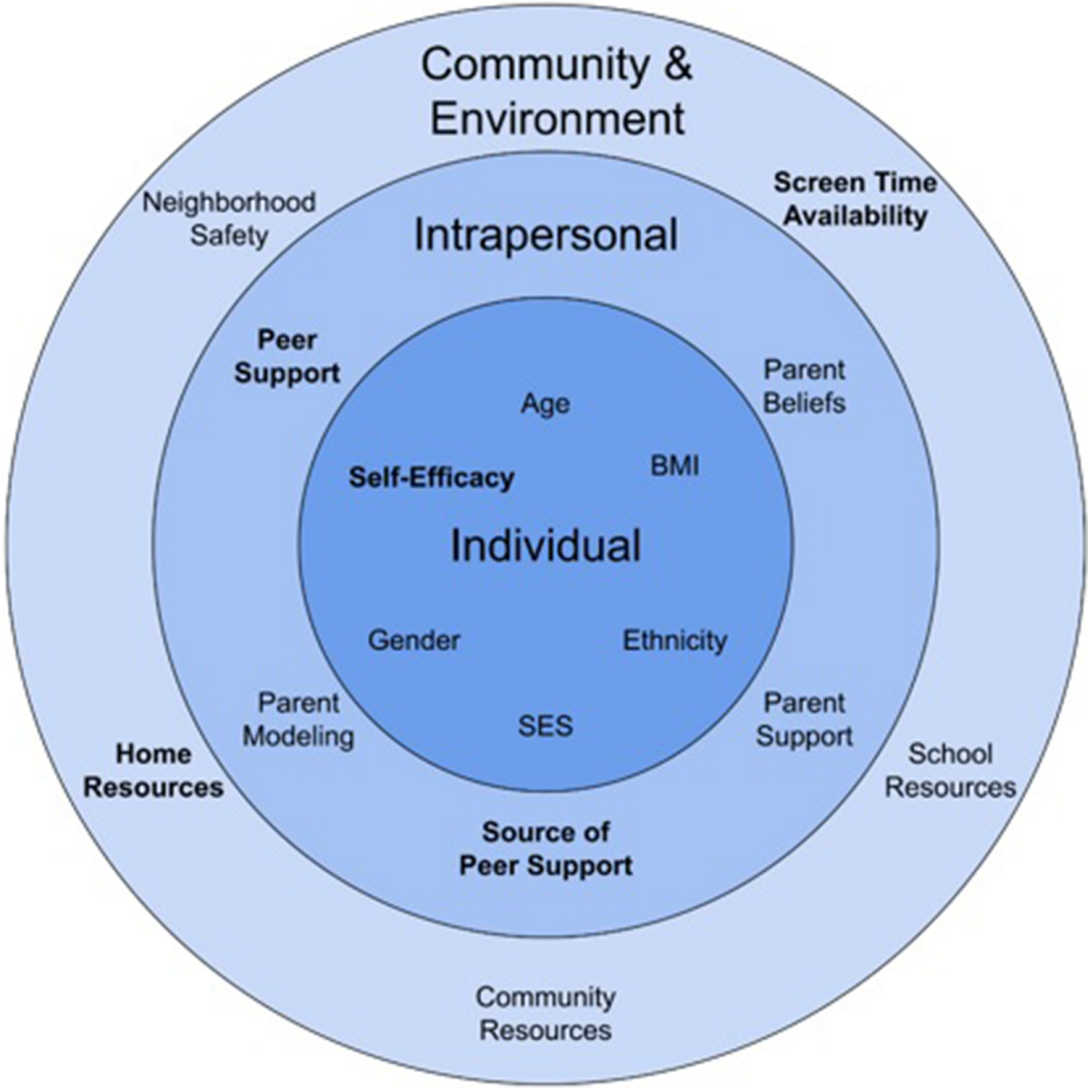
Impacting young children's physical activity; domains that are known and those that are underdeveloped^1^. ^1^Variables that are bolded indicate data gaps.

Utilizing the lens of the socio-ecologic model, we identified several contributors that impact young children's PA from a personal to an environmental level. However, we also discovered a few common gaps in the literature, depicted in Figure 1, that require further investigation. We feel the identified data gaps and suggestions for future research can inform potential interventions to improve PA in young children.

## 3 Identified data gaps

While self-efficacy is a robust construct associated with older children's PA levels, there is a deficit in the literature to understand how this could affect young children's PA behaviors (34). Self-efficacy, defined as an individual's confidence in their ability to fulfill a task, is typically evaluated in older children with qualitative measures, such as self-report surveys that don't lend themselves for use with preschoolers (35). While previous studies have established self-efficacy as a contributor of PA in older ages, both the construct and impact of self-efficacy on PA remain inadequately defined for preschoolers (35). Furthermore, the evolution of self-efficacy in relation to PA over time remains unclear. It is likely that self-efficacy is not a fixed variable as children age and may have varying impact on PA. It is possible that PA self-efficacy is influenced by other contributors, such as SES, gender, and social influence and may change dynamically over time in ways not yet well understood. To understand complex interactions, different types of research and research analytics would be needed.

The impact of screen use on PA in young children represents another gap in existing PA research, especially as the types of screens that young children use has increased dramatically (36). Recent research suggests that 95% of two-year-olds watch up to 15 h of television and videos in a week (37). While current data has begun to shed light on screen time in this population, we lack consistent evidence on the relationship between screen time and PA in preschoolers. Findings in older children are largely inconclusive or demonstrate a small inverse relationship (38, 39). Qualitative studies in preschool children have suggested that peers have the greatest influence on screen time outside of the home, while parents are the primary contributor of screen use at home (40, 41). Research in preschool populations up to now has focused on television exposure. However, screen use has become dynamic and includes televisions, laptops, desktops, tablets, and cellphones. As screens gain greater prevalence in our daily lives, preschoolers have greater exposure through their parents, siblings, friends, and care settings. Preschool screen access reflects their caretakers’ habits and is thus highly dependent on family culture and structure, childcare policies, peer social influence, and many other factors. Current research is insufficient to examine characteristically sporadic exposure to screens as it relies on parent estimates of screen exposure. Future research must examine this crucial data gap in preschool populations.

Many published studies examine linear relationships with occasional interactions between influencing variables. However, behavior is often shaped by complex interactions (42). For example, gender and access to outdoor PA resources contribute to PA levels, yet traditional research methodology overlooks the nuanced relationships between contributors. While existing research highlights children's preferences for gender-specific toys, little research has explored how different genders engage with home outdoor play equipment (43). Additionally, research often focuses on outdoor play habits in early childhood education centers rather than the home outdoor play environment. The home outdoor play environment may offer distinct stimuli for PA compared to childcare settings, given that, at home, familial engagement takes precedence over peer interaction. This may alter the impact of gender on PA, modified by home environment and family modeling. Ultimately, research has not investigated the interaction between gender and the home outdoor play environment in relation to preschoolers’ PA. Another example of complex interactions includes screen access in multiple settings, gender, and social environment (presence of peers, siblings, parents, etc.). Little to no empirical evidence currently exists to identify whether these (or other) pathways drive the negative influence of screens on PA, let alone what combination of these pathways are operating at what strength across time and across individuals.

An additional type of research gap that we identified is the relative influence of contributors. For example, while social influence plays a crucial role in children's PA, its impact may vary significantly depending on the originating social sphere. We have conceptualized potential sources of social influence, including family and home, neighborhood, school, childcare or daycare, and formal PA social networks. Within our target age group, we anticipate that familial social influence would exert the greatest impact as prior literature asserts a significant correlation between parental support and MVPA in children (44). Outside of the home, social connections that support PA correlate positively with increased PA in older children (45). While the literature identified the importance of social influences, the relative impact of those social influences and how they interact to affect young children's PA is unknown. For example, how much peer or sibling social influence would be necessary to result in changes in preschooler PA? While existing evidence would suggest that familial social influence on PA is most powerful at this age, there is insufficient evidence to be certain or to compare this response to that of other social influences.

Further research is needed to fill these gaps. With so many crucial factors missing from the equation, it is difficult to advance in the field. Below, we suggest several investigations that we believe may aid in closing these gaps.

## 4 Discussion

### 4.1 Suggestions for future research directions

Early childhood is a critical developmental stage for establishing behaviors including PA. While we have evidence of contributors that influence these behaviors in young children, there is much we do not yet know. Below, we suggest several potential research directions to build a stronger foundation in the field of early childhood PA.

Positive PA self-efficacy is an important contributor of PA in school-aged children, but its impact is not yet understood in preschoolers (35). Existing research utilizes self-scoring measurement tools, such as the Self-Efficacy Questionnaire for Children (SEQ-C) and the Children's Self-Efficacy Scale, to evaluate self-efficacy in adolescents (46). Few studies have evaluated the validity of self-efficacy measurement tools in preschool children. Recently, researchers in Italy successfully evaluated self-efficacy and enjoyment of PA in preschoolers using pictorial scoring tools (47). After validation, conducting a cross-sectional study on self-efficacy and daily amounts of PA in preschool-aged children could be an important foundational approach. PA levels could be measured by accelerometers over the course of a week, then evaluated for correlation between self-efficacy and PA. While simple in design, we believe this could evaluate self-efficacy as a potential contributor of PA in preschool children, as well as understanding how the construct of self-efficacy emerges in young children.

Another crucial area for future research is the effect of screen time on PA in young children. While research in older populations has largely been inconclusive or exhibits a slightly negative correlation, little is known about if or how it affects PA in preschool children. Given that screen time is incredibly dynamic with greater access through portable devices, we advocate that an observational study would be best to evaluate all types of screen use in this population. Accelerometer data could be used to quantitatively evaluate PA. This study may be helpful in elucidating an interaction between multiple types of screen time and PA in young children, as previous research has primarily focused on the use of parent reports which often underestimate screen use as well as primarily evaluating stationary screen sources such as TVs. This observational study would allow for a true evaluation of evolving screen use, as screens have become integral in the ways we live, work, and play.

Much of the published research on childhood PA uses a methodology and analytic approach that is cross-sectional or linear in nature or relies on assumptions about subject independence, even though researchers have broadly begun shifting to a conceptualization of PA behavior as the result of a complex and adaptive system of factors (48, 49). At present, there is an overall lack of data on the overlapping, interacting, dynamic, and heterogeneous sources of influence on a child's PA. A potential study to bridge this gap is an observational study of preschool-aged children's social interactions across their childcare settings and homes and its correlation with objective PA data. We suggest observing social interactions of preschool children both in childcare settings and at home during free play hours. In doing so, we could compare the PA levels across physical and social settings for children to determine a degree of influence from each setting, comparing the impact of home PA influences to those of childcare. This could be extended with analyses that explicate how self-efficacy levels or trajectories are affected by social environments (e.g., PA of peers, parents, or role models). This could allow insight into the likely complex interplay between children's interactions, cognition, and behaviors that can shed light on the dynamic nature of social influence on PA. In addition, it may be possible to evaluate the weight of social influence on PA in preschool-aged children across children (e.g., by gender or age) or various settings (e.g., how it is moderated by aspects of the built environment). Analyzing complex interactions can be challenging and would require differing analytic techniques (50). Ultimately, these types of studies would allow for a more comprehensive understanding of PA in preschoolers.

### 4.2 Conclusion

While previous investigations have contributed to many factors influencing PA in preschoolers, there is more to be uncovered. Of the gaps we identified, we note a pronounced deficiency in research that examines the dynamic nature of PA. We identified crucial gaps in extant literature and proposed options for future research that may guide future PA interventions, ultimately improving PA in young children.
